# Recurrence Quantification Analysis for Scene Change Detection and Foreground/Background Segmentation in Videos

**DOI:** 10.3390/jimaging11040113

**Published:** 2025-04-08

**Authors:** Theodora Kyprianidi, Effrosyni Doutsi, Panagiotis Tsakalides

**Affiliations:** 1 Foundation for Research and Technology—Hellas, 70013 Heraklion, Greece; kyptheod@ics.forth.gr (T.K.); tsakalid@ics.forth.gr (P.T.); 2 Computer Science Department, University of Crete, 71500 Heraklion, Greece

**Keywords:** recurrence quantification analysis (RQA), dynamic video processing, scene change detection, foreground/background segmentation, video analysis

## Abstract

This paper presents the mathematical framework of Recurrence Quantification Analysis (RQA) for dynamic video processing, exploring its applications in two primary tasks: scene change detection and adaptive foreground/background segmentation. Originally developed for time series analysis, Recurrence Quantification Analysis (RQA) examines the recurrence of states within a dynamic system. When applied to video streams, RQA detects recurrent patterns by leveraging the temporal dynamics of video frames. This approach offers a computationally efficient and robust alternative to traditional deep learning methods, which often demand extensive training data and high computational power. Our approach is evaluated on three annotated video datasets: Autoshot, RAI, and BBC Planet Earth, where it demonstrates effectiveness in detecting abrupt scene changes, achieving results comparable to state-of-the-art techniques. We also apply RQA to foreground/background segmentation using the UCF101 and DAVIS datasets, where it accurately distinguishes between foreground motion and static background regions. Through the examination of heatmaps based on the embedding dimension and Recurrence Plots (RPs), we show that RQA provides precise segmentation, with RPs offering clearer delineation of foreground objects. Our findings indicate that RQA is a promising, flexible, and computationally efficient approach to video analysis, with potential applications across various domains requiring dynamic video processing.

## 1. Introduction

Recent statistics indicate that the volume of data captured, transmitted, or stored worldwide each day is approximately 149 zettabytes, which is double the amount recorded in 2021 [1]. Furthermore, videos account for the majority of internet data traffic, representing over 50% of the total [2]. Therefore, there is an urgent need to enhance the algorithms currently employed for video analysis, understanding, and compression. Traditionally, videos are captured as a series of frames that contain significant spatial and temporal redundancy. Consequently, for a wide range of tasks, including scene change detection, video segmentation, object detection/tracking, and video compression, it is necessary to conduct extensive comparisons between consecutive frames to minimize redundancy and extract key information from the visual scene. However, this frame-based processing significantly increases computational costs, leading to energy-intensive algorithms that are inefficient, particularly for devices with limited battery life.

In the past decade, event-based technology has undergone extensive research and development to dynamically capture and process visual information in a way that mimics the neural architecture of the human eye and our perception of the world. This technology operates on the principle that crucial information is represented by rapid changes, referred to as “events”, inspired by the spiking behavior of biological neurons. Event-based technology has found widespread application in sensors [3], neuromorphic hardware [4], and spiking neural networks [5], which function similarly to artificial neural networks while ensuring energy efficiency.

In this work, we present an alternative method for dynamic video stream processing using Recurrence Quantification Analysis (RQA). Originally developed for time series analysis to detect recurrent patterns, RQA leverages the concept of recurrence, which parallels redundancy in image and video processing—spatiotemporal redundant information refers to data that recurs over time. The application of RQA to video streams was first introduced in [6]. Here, we enhance the mathematical framework of RQA and explore its properties in two key applications: (i) scene change detection and (ii) adaptive foreground/background segmentation over time. Our experiments demonstrate that RQA is computationally efficient and robust, outperforming state-of-the-art deep learning methods in scene change detection. Furthermore, its application to adaptive foreground/background segmentation shows promising results, enabling motion detection approaches akin to event-based processing.

Detecting scene changes in videos is a fundamental step in applications related to visual information retrieval and scene understanding. Over time, various techniques have been developed to dynamically identify these transitions, broadly categorized into traditional methods and deep learning-based approaches. Traditional scene change detection methods primarily rely on measuring similarity between consecutive frames within a sliding window [7], handcrafted features such as color, histograms, and image gradients [8], or pixel-level comparisons, global histograms, block-based histograms, and motion-based histogram techniques [9]. More recent research has shifted towards deep learning (DL) approaches, utilizing Support Vector Machines (SVMs) [10] and Convolutional Neural Networks (CNNs) either to extract relevant features or to train models for scene change prediction. Additionally, Long Short-Term Memory (LSTM) networks have been employed to aggregate temporal information from CNN-extracted features, enabling more robust scene classification [11] and Variational Auto-Encoders (VAE) have been used to identify significant changes in the dynamic scenes of maritime video data [12].

The structure of this paper is as follows: Section 2 provides a brief overview of RQA methods, initially developed for time-series analysis and subsequently adapted for images. Section 3 presents the mathematical framework of the proposed approach, enabling the application of RQA to video streams. The experimental results are discussed in Section 4, which is divided into two parts: the first focuses on the materials, methods, and results when RQA is applied to scene change detection, while the second addresses the materials, methods, and results for foreground/background segmentation. This section also includes all relevant discussions of the experimental findings. Finally, Section 5 summarizes the conclusions of this work.

## 2. Recurrence Quantification Analysis: Theoretical Framework

Recurrence Quantification Analysis (RQA) is a technique for analyzing time series data by examining the recurrence properties of dynamical systems. Its primary aim is to uncover patterns and dynamics within the system, including determining the optimal embedding dimensionality *D* and time delay τ for reconstructing the phase-space trajectory, as proposed by Takens [13]. This reconstructed phase space allows for the identification of recurrence structures, enabling the analysis of whether the original signal exhibits recurrence.

This phase-space reconstruction captures the underlying dynamics of the system by embedding the observable time series $x=(x_{1},x_{2},\ldots ,x_{n})$ into a higher-dimensional space. Specifically, the reconstructed trajectory is represented as a set of vectors in the form:

(1) $$X=\left[\begin{array}{c} X_{1}\\ X_{2}\\ \vdots \\ X_{n-(D-1)\tau } \end{array}\right]=\left[\begin{array}{cccc} x_{1} & x_{1+\tau } & \cdots & x_{1+(D-1)\tau }\\ x_{2} & x_{2+\tau } & \cdots & x_{2+(D-1)\tau }\\ \vdots & \vdots & \ddots & \vdots \\ x_{n-(D-1)\tau } & x_{n-(D-1)\tau +\tau } & \cdots & x_{n}, \end{array}\right]$$

where X comprises a set of vectors $X_{i}$ generated by starting at an initial point and selecting D−1 consecutive points with a time offset of τ. This technique ensures that the reconstructed dynamics preserve the original system’s topological properties, allowing for the analysis of recurrence structures. By studying the recurrence patterns within this reconstructed space, one can gain insights into the system’s stability, periodicity, and chaotic behavior, even with a single observable variable.

To illustrate recurrence within dynamical systems Eckmann et al. [14] introduced the Recurrence plots (RP). In the recurrence plot (RP), recurrent points are displayed in black, while non-recurrent points are shown in white. The recurrent points are defined by all pairwise distances $\left|\right|X_{i}-X_{j}\left|\right|$ between vectors $X_{i}$ and $X_{j}$. If the distance is smaller than a threshold ε then the point $R_{i,j}$ in RP is considered recurrent, following the formula:

(2) $$R_{i,j}=\Theta (\varepsilon -||X_{i}-X_{j}||),$$

where Θ(x) represents the Heaviside step function, which equals 0 for x<0 and 1 for x≥0. The selection of the threshold ε is critical for identifying recurrences within the RP. Striking the right balance is essential: ε must be small enough to maintain precision while still ensuring an adequate number of recurrences and recurrent structures [15]. Since there is no universal guideline for choosing ε, its value should be tailored to the specific application and experimental conditions.

The RP is symmetric, thus the main diagonal line inherently comprises recurrent points, as $R_{i,i}=1$ by definition. To be able to quantify the RPs, various metrics for recurrence quantification analysis have been developed [16,17,18]. Unclear dynamical behaviors in the original time series can be revealed by the measures of RQA. Some of these metrics are the recurrence rate (RR), which indicates the percentage of recurrent points in the RP, the Determinism (DET), which measures the recurrent points present in diagonal structures, the maximal diagonal length (Lmax) excluding the main diagonal, and the entropy (ENT), which quantifies the signal complexity in bits per bin.

### RQA for Image Analysis

An extension of the one-dimensional method of the RPs and their quantification to higher dimensions was proposed by Marwan et al. [19] and later by Wallot et al. [20]. The extension of the method to higher dimensions can serve as an effective approach to manage and analyse multiple features [21]. Their method was applied in 2D images to reveal and quantify recurrent structures within images. For a d-dimensional system, they defined an n-dimensional RP using the following formula:

(3) $$R_{i,j}=\Theta (\varepsilon -||X_{i}-X_{j}||),$$

where i is the d-dimensional coordinate vector and $X_{i}$ is the phase space vector at the location given by the coordinate vector i. In other words, the vector i stands for the coordinates $\left(i_{1},i_{2}\right)$ where $i_{1}$ and $i_{2}$ are the row and column indexes of the input image, respectively. The dimension of the resulting RP is n=2∗d, and even though it cannot be visualized, its quantification is still possible by computing the RQA measures in the n-dimensional RP. In the one-dimensional approach, the RP features a main diagonal line. Similarly, in the n-dimensional RP, there are diagonally oriented structures of d dimensions. When the method’s input is a 2D image, the resulting RP is a 4D plot, and slices of that RP can be visualized as 3D subsections of the 4D RP.

The authors in [19] showed that using this extension typical spacial structures could be distinguished employing recurrences. They applied this method to biomedical images, to evaluate the bone structure from CT images of the human proximal tibia. The authors in [22] introduced a method that utilizes a fuzzy c-means clustering approach and RQA measures (FCM-RQAS) for extracting image features, which can then be employed for training a classifier.

## 3. Proposed Methodology

**Figure 1:**
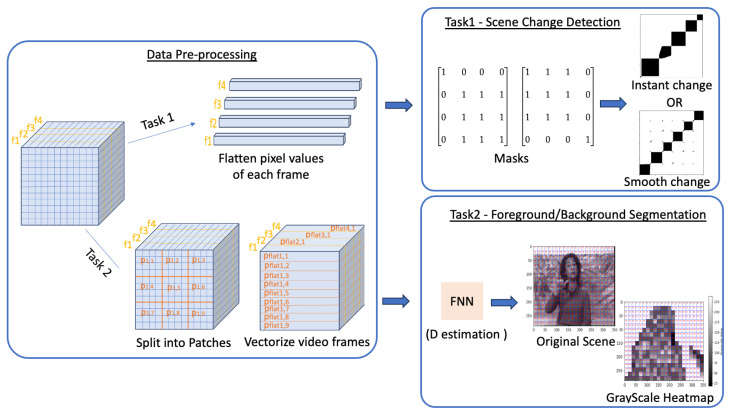
Proposed Methodology. The methodology consists of three distinct blocks: the first involves data pre-processing, which is essential for generating the vectors processed by RQA. The second block focuses on scene change detection, while the third block addresses foreground/background segmentation.

The general pipeline of this work is illustrated in Figure 1. The video stream undergoes pre-processing to generate the necessary vectors, which are then processed using RQA and compared to construct the RP. In the top-right section, we outline the key processing steps involved in scene change detection. This process relies on masks to identify frames where the scene content changes. In the bottom-right section, we illustrate the methodology for segmenting the visual scene into foreground and background. A crucial step in this process is applying the FNN algorithm, which estimates the D parameter for each patch. This parameter is then used to generate the grayscale heatmap.

### 3.1. RQA for Foreground/Background Segmentation in Videos

In our previous work, we introduced an extension of the RQA method to handle higher-dimensional data. In this study, we build upon that initial approach by enhancing its mathematical framework and improving its representation. Consider a video stream consisting of *m* frames, where each frame has dimensions M×N pixels, as defined below:

(4) $$f=(f_{1},f_{2},f_{3},\ldots ,f_{m}),$$

where f represents the video and $f_{i}$ denotes the *i*-th frame. Each frame is divided into smaller patches, with the patch size $\left(M_{p},N_{p}\right)$ determined by the frame dimensions (M,N). We assume no overlap between patches, thus, within a frame of size (M,N), there are $M/M_{p}\times N/N_{p}$ non-overlapping patches. Equation (5) illustrates the first frame of the video, $f_{1}$, divided into patches $p_{i,j}$, where *i* denotes the frame number, and *j* identifies the patch within that frame:

(5) $$f_{1}=\left[\begin{array}{cccc} p_{1,1} & p_{1,2} & \cdots & p_{1,N/N_{p}}\\ \vdots & \vdots & \ddots & \vdots \\ p_{1,\left(M/M_{p}-1\right)*\left(N/N_{p}\right)+1} & p_{1,\left(M/M_{p}-1\right)*\left(N/N_{p}\right)+2} & \cdots & p_{1,\left(M/M_{p}\right)*\left(N/N_{p}\right)} \end{array}\right]$$

For two representative patches, $p_{1,1}$ and $p_{1,\left(M/M_{p}\right)*\left(N/N_{p}\right)}$, corresponding to the $M_{p}\times N_{p}$ top-left and bottom-right pixels of frame $f_{1}$, respectively, the content of these patches is given as follows:

(6) $$p_{1,1}=\left[\begin{array}{ccc} x_{1,1} & \cdots & x_{1,N_{p}}\\ \vdots & \ddots & \vdots \\ x_{M_{p},1} & \cdots & x_{M_{p},N_{p}} \end{array}\right],\;p_{1,\left(M/M_{p}\right)*\left(N/N_{p}\right)}=\left[\begin{array}{ccc} x_{M-M_{p}+1,N-N_{p}+1} & \cdots & x_{M-M_{p}+1,N}\\ \vdots & \ddots & \vdots \\ x_{M,N-N_{p}+1} & \cdots & x_{M,N} \end{array}\right].$$

Here, $\left(x_{1,1},\ldots ,x_{M,N}\right)$ represent the pixels of the frame, with coordinates (1,1) for the top-left corner and (M,N) for the bottom-right corner.

Each patch of size $M_{p}\times N_{p}$ is finally flattened resulting in a row vector of $\left(M_{p}\times N_{p}\right)$ pixel values, as shown in Equation (7)

(7) $$\begin{array}{cc} p_{{\mathrm{flat}}_{1,1}} & =\left[\begin{array}{c} x_{1,1},x_{1,2},\ldots ,x_{M_{p},N_{p}} \end{array}\right]\\ p_{{\mathrm{flat}}_{1,\left(M/M_{p}\right)*\left(N/N_{p}\right)}} & =\left[\begin{array}{c} x_{M-M_{p}+1,N-N_{p}+1},x_{M-M_{p}+1,N-N_{p}+2},\ldots ,x_{M,N} \end{array}\right] \end{array}$$

Next, we have to determine the RQA parameters, i.e., the embedding dimension (*D*) and the time lag (τ), and then calculate vector *V* (Equation (8)) for each patch through the frames of the video. We obtain as many vectors *V* as the number of patches the video is divided, then we apply Equation (2) to obtain the RP for each patch and we observe the recurrences of each patch over time (frames).

(8) $$V=\left[\begin{array}{c} V_{1}\\ V_{2}\\ \vdots \\ V_{m-(D-1)\tau } \end{array}\right]=[\left[\begin{array}{c} p_{{\mathrm{flat}}_{1,1}}\\ \vdots \\ p_{{\mathrm{flat}}_{1+(D-1)\tau ,1}} \end{array}\right]\;\left[\begin{array}{c} p_{{\mathrm{flat}}_{2,1}}\\ \vdots \\ p_{{\mathrm{flat}}_{2+(D-1)\tau ,1}} \end{array}\right]\;\cdots \;\left[\begin{array}{c} p_{{\mathrm{flat}}_{m-(D-1)\tau ,1}}\\ \vdots \\ p_{{\mathrm{flat}}_{m,1}} \end{array}\right]{]}^{T}$$

### 3.2. RQA for Scene Change Detection

**Figure 2:**
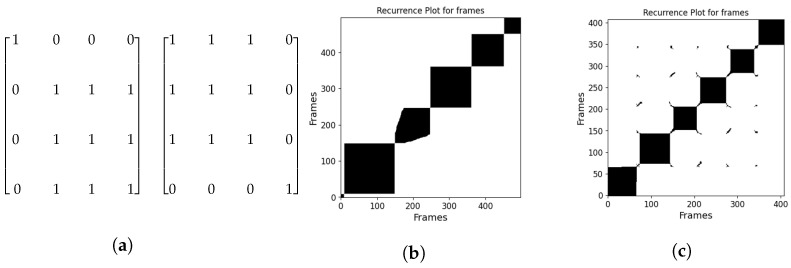
(**a**) (**Left**) This mask determines the first frame of the scene. (**Right**) This mask indicates the last frame of the scene. (**b**,**c**) Recurrence Plot for whole frames each black block defines a scene, for (**b**) the scene change is instant and for (**c**) it is gradual.

When RQA is used for scene change detection, the video frames are not divided into smaller patches but run through whole frames. We have a resulting RP for each video. The RP is then scanned with a mask to identify the frames where a scene change occurs. We applied two masks, one to determine the starting frame of a scene and one for the ending frame of a scene. The mask shown in Figure 2a is named mask3 because it has three 1s in the square of 1s. As explained in the experimental results section, we tried the same mask but with different sizes of squares of 1s, and the mask that gave the best results was mask5 in terms of F1 score of our method compared to the ground truth for the data used.

One of the main parameters of RQA is the threshold ε. For scene change detection ε is set to low PSNR values, since the whole frame is analyzed and we want to detect a major difference between two frames. The masks we are using identify the starting and ending frame, by finding the edges of the black blocks along the diagonal. Figure 2b presents the RP for a video with approximately 500 frames, featuring sharp transitions between scenes. Each black block along the diagonal represents a scene, indicating that this video consists of five consecutive scenes. In contrast, Figure 2c displays the RP for another video with smooth scene transitions. Here, discontinuities appear between the black blocks representing six different scenes, resulting from the mask’s structure, which struggles to accurately capture the scene changes.

### 3.3. Parameter Settings

#### 3.3.1. Tuning the Threshold ε

The choice of threshold ε is crucial and depends on the application. In video data analysis, identifying recurrent patches within frames is essential. However, the Euclidean distance between vector pairs $V_{i},V_{j}$ lacks significance in image processing and was ineffective for selecting ε. To address this, image quality metrics such as Peak Signal-to-Noise Ratio (PSNR) and Structural Similarity Index (SSIM) were introduced to establish a meaningful ε. Instead of computing all pairwise Euclidean distances, pairwise PSNR or SSIM values are calculated and stored in a symmetric matrix. Due to the computational cost of SSIM, the PSNR-based approach was chosen, allowing for the identification of recurrent patches that satisfy the recurrence plot (RP) equation for a given $\varepsilon_{\mathrm{PSNR}}$.

(9) $$R_{i,j}=\Theta \left(\varepsilon_{\mathrm{PSNR}}-\frac{1}{\mathrm{PSRN}(V_{i},V_{j})}\right),$$

(10) $$\mathrm{PSNR}=10\cdot \log_{10}\left(\frac{{\mathrm{MAX}}^{2}}{\mathrm{MSE}(V_{i},V_{j})}\right),$$

where $\varepsilon_{\mathrm{PSNR}}$ is the threshold value using the PSNR, MAX is the maximum pixel value found in the frame (i.e., for a grayscale image this value is 255), and MSE is the mean square error between the pixels of the original and the reconstructed image.

#### 3.3.2. Selection of the Dimensionality *D*

The embedding dimension *D* is another crucial parameter in RQA. The False Nearest Neighbors (FNN) method, introduced by Kennel et al. [23], is used to determine the optimal *D*. This approach evaluates the change in distance between neighboring points in phase space as *D* increases. If embedding in a higher dimension significantly alters the distance between neighboring points, they are considered false neighbors. The optimal *D* is reached when no further changes occur. We apply the FNN criterion to each patch of video frames. Starting from D=1, we reconstruct phase space vectors V and compute pairwise PSNR distance matrices for each *D*. For every row in the PSNR matrix, we track the position of the maximum PSNR value and compare it across successive embedding dimensions. If the difference exceeds a threshold $t_{\mathrm{PSNR}}$, the points are classified as false neighbors. The percentage of false neighbors is computed for each *D*, and the process is continued iteratively until stabilization is achieved.

#### 3.3.3. Estimating the Time Delay τ

The optimal time delay τ is determined as the first minimum of the Average Mutual Information (AMI) function, averaged across all data dimensions [24]. In this study, we set the time delay parameter to τ=1, meaning that frames are processed sequentially without skipping any intermediate ones, as our primary focus is on capturing motion changes.

#### 3.3.4. Tuning the Patch Size $\left(M_{p},N_{p}\right)$

Patch size is a crucial parameter, particularly for the foreground/background segmentation approach, as it significantly affects the segmentation accuracy. If the patch size is too large, the accuracy decreases because critical regions that separate foreground motion from the static background may be lost. In contrast, setting the patch size too small improves segmentation accuracy, but comes at the cost of significantly higher computational complexity. In this work, the patch size has been set to 8×8, as it provides a good balance between accuracy and computational cost. A more detailed comparative analysis of patch size is available in the Supplementary File.

## 4. Experimental Results

### 4.1. Dataset for Scene Change Detection

The use of RQA for scene change detection is evaluated in three annotated video datasets, each indicating the frame where a scene change occurs. These 3 datasets were also used by the Autoshot approach [25], which applies neural architecture search within a space that integrates advanced 3D ConvNets and Transformers. The first dataset, Autoshot [25], comprises 853 short videos, each lasting less than one minute. The second dataset, RAI [26], includes 10 randomly selected broadcast videos, each 3–4 min long, sourced from the Rai Scuola video archive and mainly featuring documentaries and talk shows. The third dataset is the BBC Planet Earth documentary series [27], consisting of 11 long videos, each approximately 50 min long.

### 4.2. Results for Scene Change Detection

We applied our method to the previously mentioned datasets: Autoshot, RAI, and BBC. Due to the sharpness of the masks used in our approach, our method is more effective at detecting abrupt scene changes compared to gradual transitions. We tested various values of ε ranging from 12 to 20, finding that the optimal values for F1 score were $\varepsilon_{\mathrm{PSNR}}=15$ for Autoshot and RAI, and $\varepsilon_{\mathrm{PSNR}}=18$ for the BBC dataset. Additionally, as shown in Table 1, the best results in terms of F1 score were achieved with mask5, when comparing our method to the ground truth for the tested data.

Table 2 presents the F1 scores for the three datasets, considering both all scene changes and only abrupt scene changes. Mask5 was used for all tests, with the corresponding ε values for each dataset listed. The F1 scores for abrupt changes alone are higher across all three datasets, demonstrating that our method more accurately detects instant scene changes. Next, we compare our method with other methods that identify scene changes. Table 3 shows the F1 scores for different video scene change detection methods across various datasets. Autoshot (2023) represents a state-of-the-art approach leveraging neural networks to analyze video data. In contrast, our proposed method, RQA, applies a mathematical framework directly to the video data. We need to highlight that despite the difference in approach, RQA has a comparable F1 score with Autoshot method. Additionally, in some cases, it outperforms other methods and/or has comparable results with them. Notably, most of the methods use neural networks, and the results for RQA highlight its effectiveness and potential. One of the most important reasons to claim that is the deep learning (DL) models require distinct training processes for each specific task, relying on separate datasets, and the knowledge gained remains task-dependent. For example, while the proposed method can simultaneously perform different tasks like foreground/background segmentation and scene change detection without any training, DL models must address these tasks individually, requiring dedicated training for each.

### 4.3. Dataset for Adaptive Foreground/Background Segmentation

This application requires short videos that feature a single scene, ideally with no simultaneous motion in the foreground and background. This means that all parts of the frame have motion, making it difficult for our method to distinguish between the two categories. UCF101 dataset [34] is a large dataset of human action, consisting of 101 action classes and more than 13k short clips, making it a very suitable video collection to evaluate our method. DAVIS dataset (Densely Annotated VIdeo Segmentation) [35], consists of 50 high-quality full HD video sequences that cover common video object segmentation challenges such as occlusions, motion blur, and appearance changes. The videos used in this work and the result can be found in the following url https://github.com/dwrakyp/MDPI_videos_results.git (accessed on 30 January 2025).

### 4.4. Results for Adaptive Foreground/Background Segmentation

**Figure 3:**
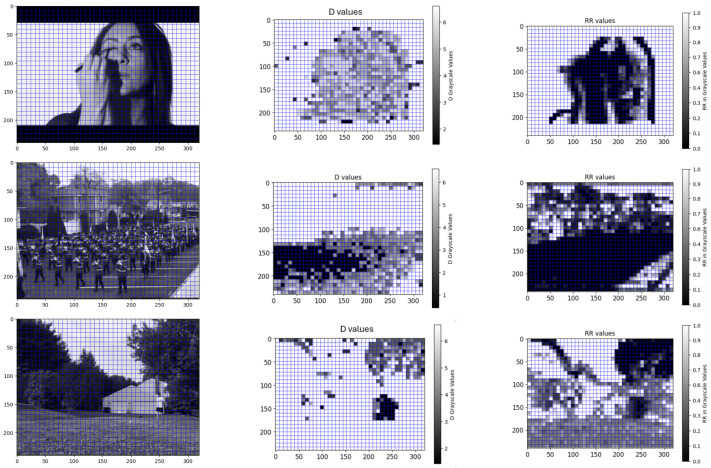
Results on the makeup video (**top** row), the parade video (**middle** row), and the ball video (**bottom** row). (**left** column) Random frame which is split into patches of a size 8 × 8. (**middle** column) Grayscale heatmap generated for the *D* values. (**right** column) Grayscale heatmap based on the RR values.

We analyzed several videos from the UCF101 dataset [34] and the DAVIS dataset [35], selecting three videos from the UCF101 dataset to showcase the results of our RQA analysis. The first video, titled ’make-up’ and shown in Figure 3 (top), features a stable camera capturing a girl applying make-up with a stationary background and motion limited to the foreground. The second video, ’parade’, illustrated in Figure 3 (middle), also uses a stable camera, focusing on a road where a parade is passing by, characterized by strong foreground motion (the parade) and minimal background motion. The third video, ’ball’, represented in Figure 3 (bottom), depicts a stable camera view of a natural landscape with a person on the right throwing a ball; aside from minor leaf movement in the trees, the landscape remains static. These videos were chosen to demonstrate our method’s capability to handle varying types of motion: no movement, subtle movement (leaves), and significant movement (parade, make-up application).

Each video is segmented into patches, and for each patch, the optimal embedding dimension *D* is determined using the FNN algorithm, with $\varepsilon_{\mathrm{PSNR}}=35$, a value chosen experimentally. A grayscale heatmap illustrating the optimal *D* is displayed in the middle column of Figure 3. As anticipated, RQA effectively detects and highlights regions of motion in the image, categorizing them as foreground, while areas with minimal or no motion are classified as background. Furthermore, we investigated the role of Recurrence Plots (RPs), which convey critical insights derived from RQA beyond its standard features. The right column of Figure 3 presents a grayscale heatmap generated from the RP for each patch, where high-intensity values indicate background regions and low-intensity values represent foreground regions. Both heatmaps demonstrate effective foreground/background segmentation, but the RP-based heatmap is significantly more precise than the *D*-value heatmap, providing a more detailed representation of object shapes within the scene. Consequently, depending on the precision required for a given application, our method offers flexibility in achieving varying levels of segmentation accuracy.

## 5. Conclusions

In this work, we have evaluated the use of Recurrence Quantification Analysis (RQA) for video analysis, focusing on two tasks: scene change detection and adaptive foreground/background segmentation.

For scene change detection, we applied RQA to three video datasets: Autoshot, RAI, and BBC Planet Earth, comparing its performance with several state-of-the-art methods. Our experiments demonstrated that RQA achieves competitive results, particularly in detecting abrupt scene changes. We showed that with optimal parameter tuning, our method can effectively detect scene changes with high F1 scores, which are comparable to the neural network-based Autoshot method. Moreover, RQA performs robustly across different datasets, indicating its generalizability and potential for practical applications.

For adaptive foreground/background segmentation, we used videos from the UCF101 and DAVIS datasets to test our method’s ability to distinguish between foreground and background regions based on motion. We found that RQA successfully identifies regions with significant motion, such as the foreground, while static areas are classified as background. By analyzing the heatmaps of the embedding dimension *D* and Recurrence Plots (RPs), we demonstrated that our method can produce precise segmentation maps, with RPs offering clearer foreground/background distinctions. The method’s flexibility allows it to adjust to different levels of precision depending on the specific needs of the application.

Overall, RQA proves to be a promising approach for both scene change detection and adaptive foreground/background segmentation, offering high accuracy and flexibility while providing insights into the dynamics of video data. Future work can explore further refinements and the integration of RQA with deep learning-based methods to enhance its performance in more complex video analysis tasks.

## Figures and Tables

**Table 1:** F1, precision, and recall scores for different mask sizes and datasets.

	RAI			Autoshot			BBC		
	F1	PRE	REC	F1	PRE	REC	F1	PRE	REC
mask9	0.823	0.961	0.719	0.750	0.789	0.714	0.778	0.982	0.645
mask7	0.832	0.948	0.741	0.762	0.790	0.737	0.781	0.981	0.648
mask5	0.835	0.939	0.752	0.757	0.750	0.765	0.783	0.972	0.655
mask3	0.829	0.912	0.760	0.747	0.709	0.790	0.776	0.935	0.662
mask2	0.588	0.475	0.719	0.529	0.393	0.809	0.547	0.464	0.664

**Table 2:** (**a**) RQA results all scene changes, (**b**) RQA results only for instant scene change.

(a)			
	Autoshot	RAI	BBC
$\varepsilon_{PSNR}$	15	15	15
TP	1894	740	4100
FP	632	48	521
FN	581	244	747
PRE	0.749	0.939	0.887
REC	0.765	0.752	0.846
F1	0.757	0.835	0.866
**(b)**			
	**Autoshot**	**RAI**	**BBC**
$\varepsilon_{PSNR}$	**15**	**15**	**18**
TP	1748	782	4043
FP	632	48	521
FN	350	38	668
PRE	0.734	0.934	0.885
REC	0.833	0.947	0.858
F1	0.781	0.941	0.872

**Table 3:** F1 scores for different methods. Autoshot [25], DSMs [28], ST ConvNets [29], TransNet [30], TransNetV2 [31], Hierarchical clustering [32], Deep Siamese Network [33].

Method	Autoshot	RAI	BBC
Autoshot (2023)	0.841	0.971	0.955
DSMs (2018)		0.893	0.939
ST ConvNets (2017)		0.926	0.939
TransNet (2019)		0.929	0.943
TransNetV2 (2024)		0.962	0.939
RQA	0.781	0.941	0.872
Hierarchical clustering (2015)		0.720	
Deep Siamese Network (2015)			0.620

## Data Availability

This study does not generate new data; all datasets utilized are publicly available and are properly cited in the main text. The code of this work is available at the following https://github.com/dwrakyp/MDPI_videos_results.git, accessed on 30 January 2025.

## Supplementary Materials

The following supporting information can be downloaded at: https://www.mdpi.com/article/10.3390/jimaging11040113/s1, Figure S1: Results on the makeup video (top row), the parade video (middle row), and the ball video (bottom row). (left column) Random frame which is split into patches of a size 16 × 16. (middle column) Grayscale heatmap generated for the D values. (right column) Grayscale heatmap based on the RR values. Figure S2: Results on the makeup video (top row), the parade video (middle row), and the ball video (bottom row). (left column) Random frame which is split into patches of a size 32 × 32. (middle column) Grayscale heatmap generated for the D values. (right column) Grayscale heatmap based on the RR values. Figure S3: Results on the makeup video (top row), the parade video (middle row), and the ball video (bottom row). (left column) Random frame which is split into patches of a size 8 × 8 and for PSNR threshold ε = 45. (middle column) Grayscale heatmap generated for the D values. (right column) Grayscale heatmap based on the RR values. Figure S4: Results on the makeup video (top row), the parade video (middle row), and the ball video (bottom row). (left column) Random frame which is split into patches of a size 8 × 8 and for PSNR threshold ε = 20. (middle column) Grayscale heatmap generated for the D values. (right column) Grayscale heatmap based on the RR values. Table S1: Execution time for FNN and RQA for different patch sizes.
