# Supplementary material for: Recurrence Quantification Analysis for Scene Change Detection and Foreground/Background Segmentation in Videos
Source: J Imaging. 2025 Apr 8;11(4):113. doi: 10.3390/jimaging11040113 (PMC12027938; doi:10.3390/jimaging11040113)
Supplement: Supplementary file 1 [file jimaging-11-00113-s001.zip › jimaging-3479047-supplementary.pdf]

Supplementary Material for: *Recurrence Quantification Analysis for Scene change detection and Foreground/Background segmentation in videos*

Theodora Kyprianidi, Effrosyni Doutsi, and Panagiotis Tsakalides

1 Results for different RQA parameters

This supplementary material aims to enhance the representation of the experimental results and improve the understanding of the parameter settings discussed in the paper. Specifically, it focuses on two key parameters—the patch size and the threshold $\varepsilon_{\text{PSNR}}$—providing a comprehensive analysis of how these parameters were tuned in the main text.

1.1 Patch size

Patch size is a key parameter in RQA that significantly impacts segmentation accuracy. Here, we present results for the three videos from the main text using different patch sizes. While the main text displays results for a patch size of 8×8 with $\varepsilon = 35$, we now provide additional results for patch sizes of 16×16 (Figure S1) and 32×32 (Figure S2) using the same ε value. As anticipated, increasing the patch size reduces the sensitivity of RQA in foreground/background segmentation compared to smaller patch sizes, resulting in more abstract segmentation outcomes.

Additionally, in Table S1, we discuss the execution time required to determine the optimal D using the FNN algorithm, as well as the overall runtime of RQA for each patch size.

video	FNN execution time			RQA execution time		
patch size	8x8	16x16	32x32	8x8	16x16	32x32
makeup	3 min 20 sec	1 min 50 sec	4 min 50 sec	2 min 20 sec	34 sec	10 sec
parade	1 min 10 sec	64 sec	1 min 30sec	38 sec	5.7 sec	1.8 sec
ball	60 sec	22 sec	51 sec	20 sec	6 sec	1.6 sec

Table S1: Execution time for FNN and RQA for different patch sizes.

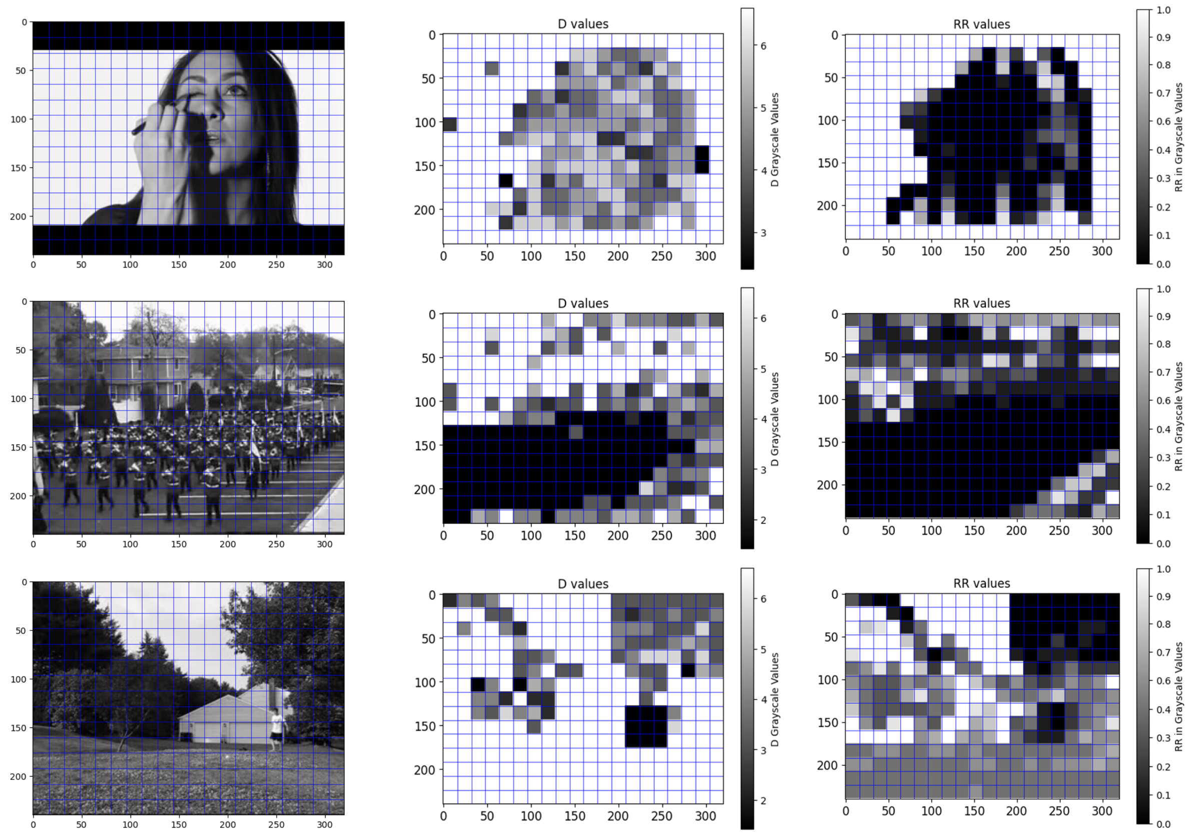

Figure S1: Results on the makeup video (top row), the parade video (middle row), and the ball video (bottom row). (left column) Random frame which is split into patches of a size 16x16. (middle column) Grayscale heatmap generated for the D values. (right column) Grayscale heatmap based on the RR values

1.2 PSNR threshold for motion segmentation

The PSNR threshold $\varepsilon_{\mathrm{PSNR}}$ is a parameter that determines the outcome of the RQA analysis for motion segmentation. Here we present the result for different values of $\varepsilon_{\mathrm{PSNR}}$, $\varepsilon_{\mathrm{PSNR}} = 45$ (Figure S3) and $\varepsilon_{\mathrm{PSNR}} = 20$ (Figure S4.A large $\varepsilon_{\mathrm{PSNR}}$ reduces the number of recurrences in the RP, resulting in a lower RR value for the patch. Thus, the heatmap of the RR values will contain more patches with a lower RR, potentially failing to correctly classify some patches as foreground. A higher $\varepsilon_{\mathrm{PSNR}}$ sets stricter criteria for identifying stable patches, leading to the misclassification of certain patches as having motion when they do not exhibit significant motion. A low $\varepsilon_{\mathrm{PSNR}}$ has the opposite effect; it increases the recurrences in the RP, leading to a higher RR value for a patch. As a result, the RR heatmap will have higher values, potentially causing some patches to be incorrectly classified as stable despite exhibiting some motion.

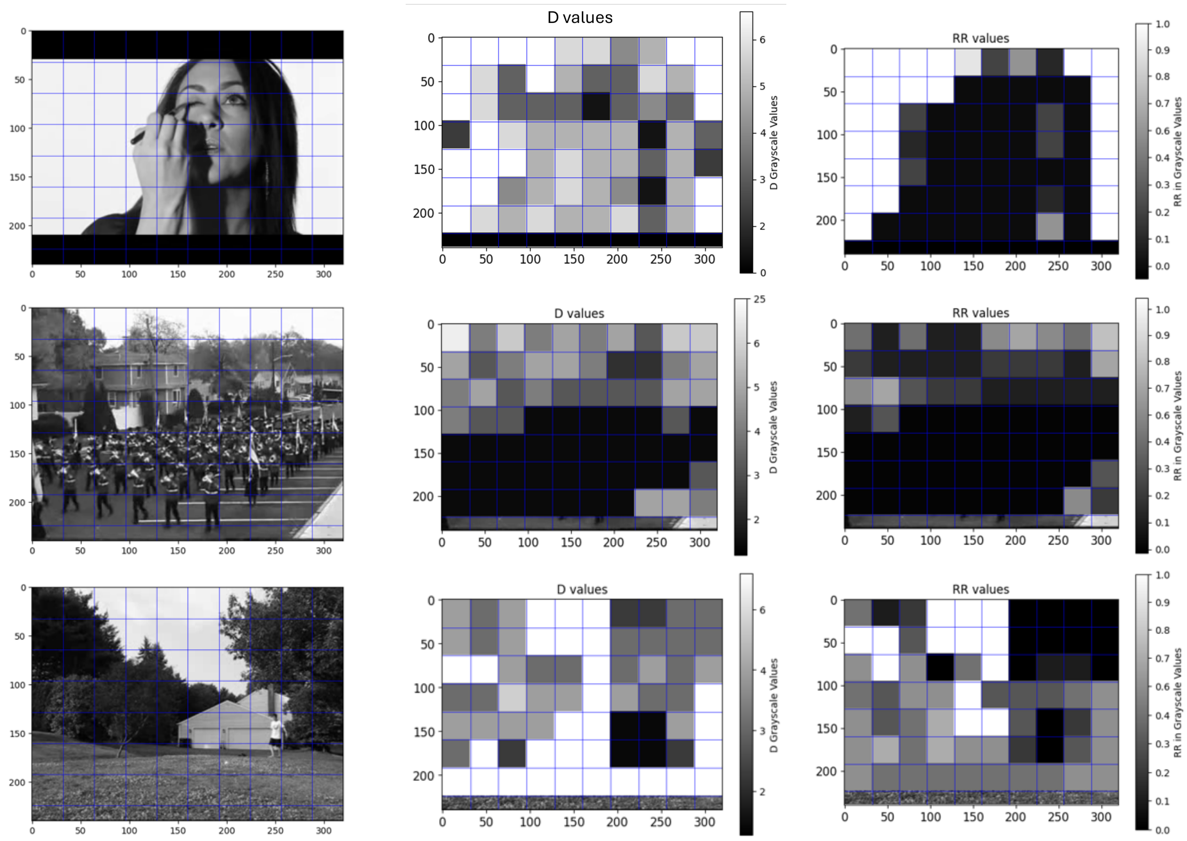

Figure S2: Results on the makeup video (top row), the parade video (middle row), and the ball video (bottom row). (left column) Random frame which is split into patches of a size 32x32. (middle column) Grayscale heatmap generated for the D values. (right column) Grayscale heatmap based on the RR values

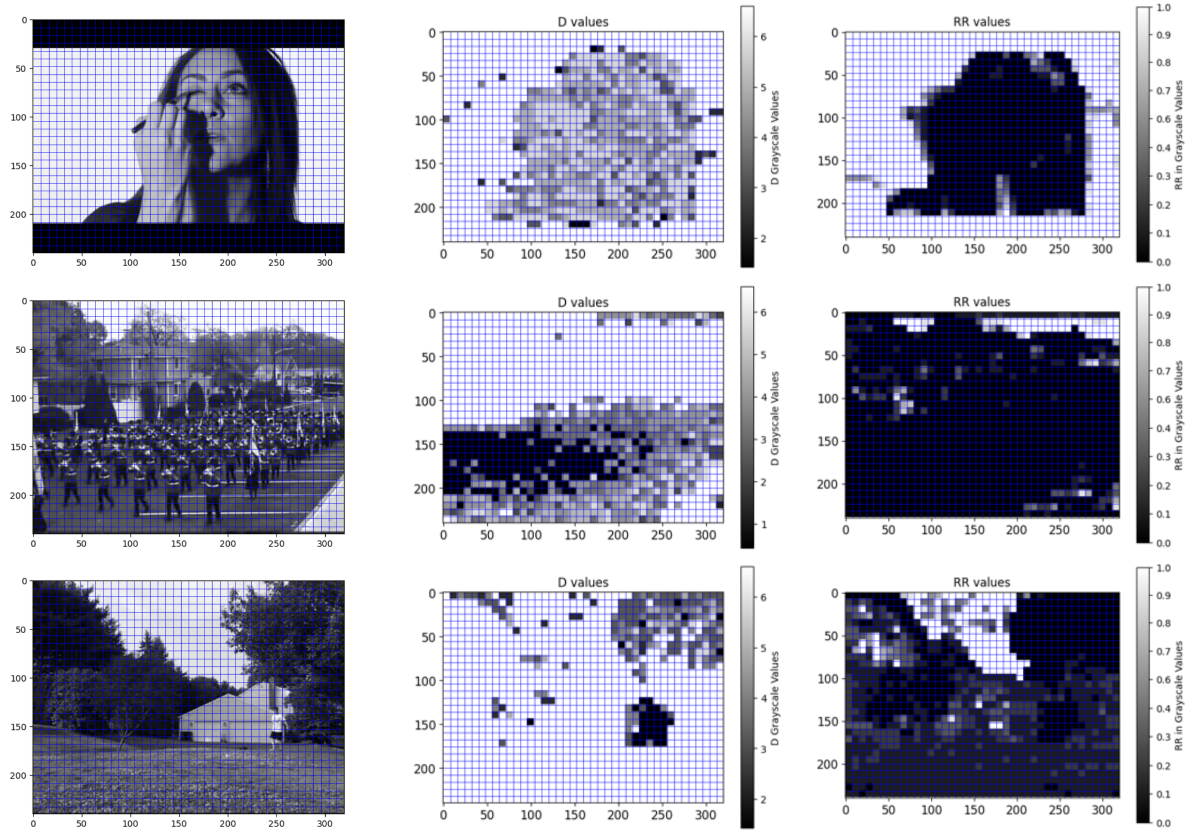

Figure S3: Results on the makeup video (top row), the parade video (middle row), and the ball video (bottom row). (left column) Random frame which is split into patches of a size 8x8 and for PSNR threshold $\varepsilon = 45$. (middle column) Grayscale heatmap generated for the D values. (right column) Grayscale heatmap based on the RR values

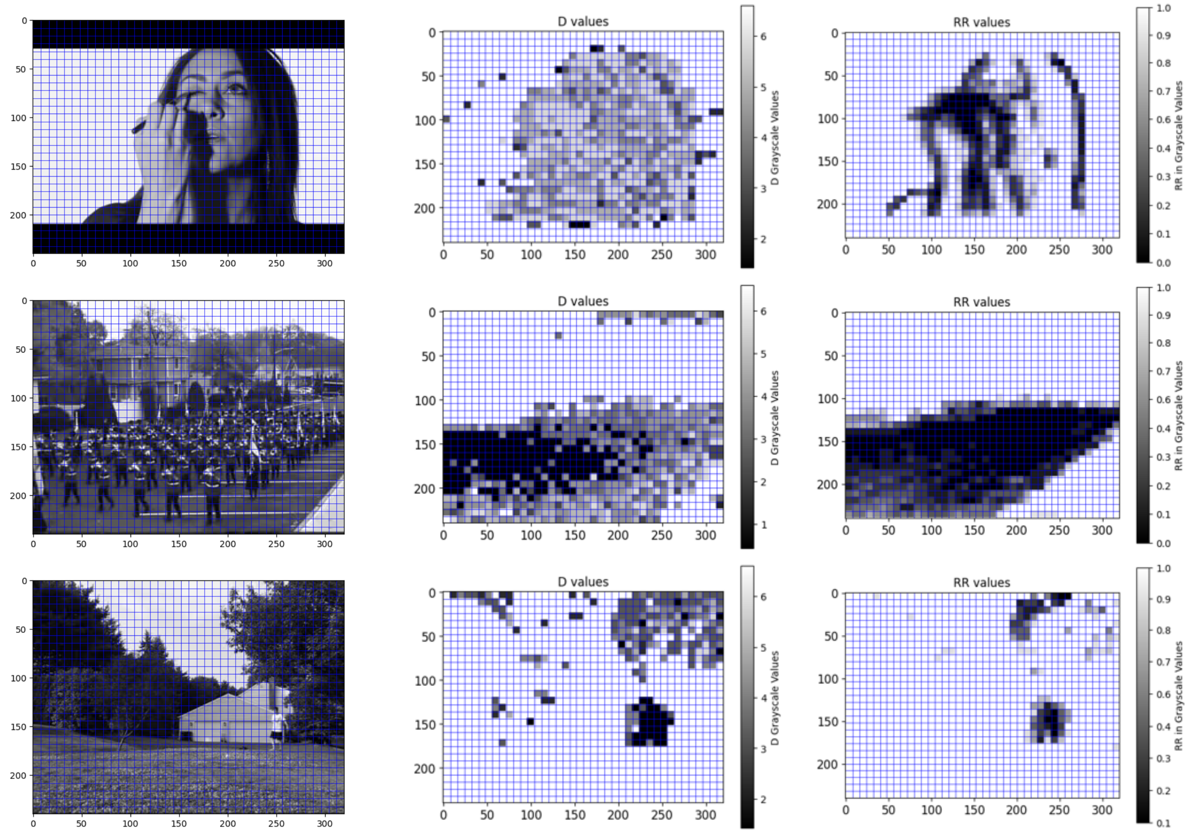

Figure S4: Results on the makeup video (top row), the parade video (middle row), and the ball video (bottom row). (left column) Random frame which is split into patches of a size 8x8 and for PSNR threshold $\varepsilon = 20$. (middle column) Grayscale heatmap generated for the D values. (right column) Grayscale heatmap based on the RR values
